# PReS-FINAL-2310: Successful rituximab treatment in a 14 year-old boy with lupus nephritis

**DOI:** 10.1186/1546-0096-11-S2-P300

**Published:** 2013-12-05

**Authors:** V Torrente-Segarra, E Iglesias, R Bou, S Ricart, MI Gonzalez, J Sanchez, L Lopez-Vives, J Antón-López

**Affiliations:** 1 Pediatric Rheumatology, Hospital Sant Joan Deu, Esplugues Llobregat, Spain

## Introduction

SLE treatment.

## Objectives

to present the case of a child with refractory lupus nephritis that responded to rituximab therapy in a systemic lupus erythematosus (SLE) child.

## Methods

we describe sociodemographic, clinical and laboratory follow-up of a 14-yo boy with lupus nephritis. Treatment was based in Euro-Lupus recommendations. We collected all data from patient's chart from our database at Hospital Sant Joan de Déu, in Esplugues Llobregat (Catalunya, Spain). From the follow-up clinical charts we collected sociodemographics, specific SLE serological profile, proteinuria level, and treatment received.

## Results

a 12-yo boy was initially visited in our Clinics due to the presence of ANA low titre (1/40) and recurrent generalized arthralgia, in association to livedo reticularis. Neither DNAds positivity nor evidence of inflammatory pattern of his arthralgia was found. Two years later (April'10) showed up with following symptoms: fever, blaschkitis, ANA 1/160, DNAds positivity (low titre), Sm positivity, hypocomplementemia, and mild plaketopenia. Active urine sediment was also found: proteinuria and hematuria (+++). We performed a complete 24 h urine test, showing 2.4 g/24 h proteinuria. SLEDAI score was 14. Patient was immmediatly admitted and a renal biopsy was performed. It showed Class IV-WHO-Lupus Nephritis (LN) with 17/24 and 0/12 activity and chronicity scores, respectively.

The patient initiated therapy with hydroxyclorochine (2 mg/kg/d), prednisone (1 mg/kg/d) and 3 cychlophosphamide pulses, and hyposodic diet; however, no improvement was observed after 2 months. Then, mycophenolate acid was added, reaching 720 mg/daily, with no improvement after another 2 months. In July '10 the patient received the first of 4 doses of rituximab (375 mg/m2/every other week). We also initiated prophylaxis treatment for P. carinii. After 2 months, proteinuria dramatically improved. Although proteinuria descended to 2 g/24 h we performed a re-biopsy in October'10 due to the persistence of the proteinuria: Class IV-WHO-Lupus Nephritis was also observed, and chronicity index was 6/24 and activity index was 4/12. All the other serological parameters were also improved. In December'10 patient reached proteinuria <500 mg/24 h, normal values in all activity and serological parameters. SLEDAI score was 0. We started to taper steroids. In the last follow-up visit patient maintained clinical remission receiving: hydroxychloroquine, mycophenolate acid, and 5 mg prednisone daily. Neither infections nor adverse events after 2 years of rituximab treatment have been observed.

## Conclusion

we describe a case of refractory LN successfully treated with rituximab. Conventional treatments fail to control LN disease. Rituximab offers an alternative for severe patients who do not respond to immunosuppressant. Rituximab seems to provide a good safety profile. Larger series are needed to confirm this data in juvenile SLE.

## Disclosure of interest

None declared.

